# Study on the reliability and validity of the Chinese version of the CMAI Scale in patients with cognitive impairment

**DOI:** 10.1002/alz70861_108676

**Published:** 2025-12-23

**Authors:** Boru Jin, Aitong Li, Huayan Liu

**Affiliations:** ^1^ The First hospital of China medical university, Shenyang, Liaoning China; ^2^ The First Hospital of China Medical University, Shenyang, Liaoning China; ^3^ Department of Neurology, the First Hospital of China Medical University, Shenyang, liaoning China

## Abstract

**Background:**

This study aims to translate and compile the CMAI scale in combination with Chinese culture, and test the reliability and validity of the Chinese version.

**Method:**

242 patients with cognitive impairment were collected in the Outpatient Department of the First Hospital of China Medical University from September 2023 to March 2025. All of them completed the Mini‐Mental State Examination(MMSE), Montreal Cognitive Assessment(MoCA), Neuropsychiatric Inventory(NPI), Clinical Dementia Rating(CDR), and the revised Chinese version of CMAI scale (CMAI‐C) through face‐to‐face interviews with patients and their caregivers. The reliability test of the CMAI‐C scale is conducted by calculating and evaluating the internal consistency, test‐retest reliability, and inter‐rater reliability of the overall and each part of the study.

**Result:**

Baseline characteristics are as follows: average age (67.76±7.84), average scores of MMSE (21.32±5.31), average scores of MoCA(15.77±5.85). 79.25% of the whole patients, 58.97% of mild cognitive impairment (MCI), 85.71% of mild, and 96.87% of moderate dementia patients suffer from agitation. The most common symptoms are negativity, hiding items, cursing others, or threatening or insulting others in words. In the reliability analysis, the Cronbach's alpha coefficient was 0.913, revealing good feasibility. 20 patients were randomly selected, and the Spearman correlation coefficient between their initial and two weeks later retest scores was calculated to evaluate the retest reliability, which was 0.879, indicating a good correlation. Also, 20 randomly selected patients were tested by two professors, and the intraclass correlation coefficient (ICC) was 0.975, indicating good reliability. On the validity analysis, the content validity was tested by CVI, with a total of 8 experts conducting reviews. The I‐CVI index was 0.875, indicating good content items, and the S‐CVI/UA was 0.917, suggesting good content validity. The concurrent validity of CMAI and NPI scales was calculated using the Spearman correlation coefficient, which was 0.700 (*p* <0.05), suggesting a significant correlation.

**Conclusion:**

Patients with cognitive impairment a at a high risk of agitation, and the occurrence of agitation increases as cognition declines. The revised Chinese version of the CMAI scale is an effective and reliable tool for assessing agitation in MCI and dementia, with high test and inter‐rater reliability.

